# Outcome Measures in intellectual disability: A Review and narrative synthesis of validated instruments

**DOI:** 10.1177/00207640241291517

**Published:** 2024-10-25

**Authors:** Mrityunjai Kumar, Indermeet Sawhney, Verity Chester, Regi Alexander, James Mitchell, Rohit Shankar

**Affiliations:** 1 St Helens and Knowsley Teaching Hospitals NHS Trust, Saint Helens, UK; 2 Hertfordshire Partnership University NHS Foundation Trust Hatfield, Hatfield, Hertfordshire, UK; 3 University of Liverpool, UK; 4 University of Plymouth, UK; 5 CIDER, Cornwall Partnership NHS Foundation Trust Truro, UK

**Keywords:** Intellectual disability, neurodevelopmental disorders, co-morbidity, outcome measures, health outcomes

## Abstract

**Background:**

Outcome measurement is essential to determine the effectiveness of health interventions and improve the quality of services. The interplay of social, individual, and biological factors makes this a complex process in the psychiatry of people with intellectual disability (PwID).

**Aim:**

Review of outcome measures which are validated in PwID

**Methods:**

A PRISMA-guided review was conducted, using a predefined criteria and a relevant word combination on four databases: EMBASE, Medline, CINAHL and PsycINFO. Each included study was examined for relevance to intellectual disability psychiatry. The psychometric data of each tool was critically assessed. Findings were narratively synthesised.

**Results:**

Of 1,548 articles, 35 met the inclusion criteria. Several outcome measures were identified relevant to intellectual disability psychiatry, including tools for challenging/offending behavior, specific neurodevelopmental/clinical conditions such as ADHD, epilepsy, and dementia however, psychometric properties, validity and reliability varied considerably. The tools identified were largely clinician rated, with a dearth of measures suitable for completion by patients or their family carers.

**Conclusion:**

Most outcome measures used for PwID lack suitable psychometric properties including validity or reliability for use within the ID population. Of importance, those with alternative expression or are non-verbal have been excluded from the research developing and reporting on measurement instruments. There is an underserved population who risk being left behind in the era of value-based medicine and increasing use of outcome measurement when assessing the effectiveness of healthcare interventions on individual and population levels. This is the first of its kind review in this area.

## Introduction

Measuring outcomes is essential to determine the effectiveness of health interventions and improving the quality of clinical services. However, despite increasing validation of outcome measures for people with intellectual disabilities (ID), they are still not routinely used in clinical practice. While the diagnostic categorization in psychiatry of ID is driven by strict nosological criteria (ICD11/ DSM 5) accompanied by robust diagnostic scales, outcome measurement in core symptoms of ID or comorbidities is limited (Thurm et al., 2020).

Health outcome is change in health status of individual, or population which can be attributed to an intervention. Measuring the change owing to the intervention is key to understanding their impact. This can be used for measurement of quality of interventions (Porter, 2010) as well as a performance measurement matrix for health systems (Jacobs & McDaid, 2010). To understand the full scope of outcomes, it is important to understand the whole life-cycle of interventions, rather than standalone interventions (Porter & Thomas, 2013).

Outcome measurement in psychiatry is complex due to a significant interplay of social, individual, and biological factors. This includes individual disorders, as well as functioning impacts on social and personal factors, quality of life, mortality, relapse, and readmission rates. There is wider array of non-health, social outcomes such as employment, housing, and other measures of wellbeing, including engagement in society, particularly in the ID population. While the overall health of people with ID has been a longstanding concern (Emerson & Hatton, 2013), an integrated health inequalities approach to address them is relatively recent. Hence, to measure the holistic impact on overall health, consideration of both health and non-health outcomes become relevant and important.

In terms of morbidity and mortality outcomes, adults with ID have been shown to have higher incidence of comorbid physical health problems, including diabetes and cardiovascular disease as compared to people without ID (Jansen et al., 2004), with much shorter life expectancy than non ID population with 63% deaths before 65 as compared to 10% in the general population (White et al., 2023). Consequently, measuring and accounting for interventions which can affect meaningful change is an urgent priority. Essentially, the key types of outcome measures are:

1. Clinician rated outcome measures (CROMs): CROMs classically have continued to remain mainstay for measurement of health status. They are mostly condition specific and one of the earliest established standardized reporting of magnitude of change in health conditions, however some CROMs can provide global impression of change.
2. Patient reported outcome measures (PROMs): PROMs measure health constructs as reported by patients or service users. They include health status information, functional status and quality of life measures. PROMs can be condition focused or generic (Black, 2013). In England, a small number of outcomes have been mandated to be measured using PROMs for surgical services, specifically piloted for knee and hip surgery since 2009 (NHSE, 2022). The Royal College of Psychiatrists has recommended use of routine outcome measures, and more recently, PROMs have become an overarching recommendation across most of community mental health services (‘NCCMH’, 2023). However, PROMs are not routinely collected in ID psychiatry.
3. Patient reported experience measures (PREMs): PREMs measure patients experience within the healthcare system and have the potential to improve service quality, and user responsiveness (Coulter, 2006; Goldstein et al., 2005). This can measure the standards and reliability of services at systems level which is an effective tool to inform policy and practice (De Rosis et al., 2020). These measure experience of the health service interaction, rather than health outcomes.
4. Carer reported outcome measures (CAROMs): CAROMs are proxy measures of functioning, quality of life or recovery for who may not be able to effectively complete such report. They have conventionally been in used in palliative care or for those cognitive impairment, and have been found to be feasible and appropriate (Seipp et al., 2022). However, in the field of ID, there are limited report of use of carer reported outcome measure beyond service evaluations such as friends and family surveys, which can support local quality improvement, but lack the scientific robustness to inform policy. The validated measures focusing on ID population such as HRQoL-IDD focus on health related quality of life (Pett et al., 2021) and SLDOM (Sheffield Learning Disability Outcome Measure) measures generic areas such as parental sense of self-efficacy, control and confidence among children with ID but not clinical outcomes (Delahunty et al., 2018).

While the public health approach to healthcare measures have evolved around both process and outcomes monitoring, some important properties for patient-based outcome measures are appropriateness, reliability, validity, responsiveness, precision, interpretability, acceptability and feasibility (Thornicroft & Tansella, 2013). They are also used as evidence of quality assurance, and value for money. While the aforementioned seven criteria are key to examining outcomes, precision, and generalizability, appropriateness are the most important criterion for patient related outcome measures to be effective (Fitzpatrick et al., 1998).

Porter and Lee (Porter, 2010) proposed a three tiered outcome measure hierarchy when determining the group of relevant outcomes for any condition or patient population They include health status achieved or retained, process of recovery and sustainability of health. While Porter’s model primarily assumes the healthcare states to be definitive and directly influencing the outcomes, it is seldom the case in most chronic disease conditions and more specifically in ID.

Health related Quality of Life (HRQL) model outlines a slightly holistic approach and considers continuum of bio-socio-psychological factors. The Cleary Wilson HRQL model moves across symptom status, functional level, general health conditions, and an overall quality of life across the domains of individual and environment (Wilson & Cleary, 1995). The model also allows for multidirectional flows and hence is more dynamic in nature. The international classification of functioning and disability model (ICF) has articulated this relationship more explicitly (‘WHO’, 2011) and conceptually is a more robust approach to developing outcome measures in ID as this takes into account contextual factors and is more relevant to impairment centric discourse.

In the clinical context, there are some validated CROMs which have informed the development of clinical or practice guidelines. Despite legal protections and policy directives, exclusion of people with ID from clinical research is common. This is despite advocacy frameworks at global levels such as World Health Organization (WHO) quality of life frameworks including a disability module. The United Nations Convention on the rights of persons with disabilities, and the Equality and Human Rights commission (ECHR) call for inclusive participation of people with intellectual disorders (Emerson & Hatton, 2013). To illustrate, Feldman (Feldman et al., 2014) reported that only 2% of randomized control trials (RCTs) included people with ID, with more recent reviews reporting psychiatric (68%), cognitive and intellectual (42%) as leading reasons for excluding clinical trials (Plosky et al., 2022). The overall impact of such exclusion has led to limited robust scientific data across newer therapeutic areas and interventions, hence impacting on lack of outcome measures development.

This scoping review aimed to detail the available outcome measures which are validated for this population to support the use of current outcome measures within psychiatric ID services and identify gaps and limitations in key areas of practice needing outcome tools.

## Methods

We used a review methodology supported by PRISMA guidelines as the area of outcome research in ID is a relatively new area of enquiry with sparse evidence base (Levac et al., 2010; Khalil et al., 2016) (supplementary information 1) Our methods included the following:

1. Identifying the research question: We aimed to identify outcome measurement instruments which have been validated in ID population. We kept the research question wide based to reflect key areas of clinical practice important to ID psychiatry. This was informed by a preliminary search at early stages. Following preliminary searches, to further refine the research question, priority mapping exercise within a select group of the Royal College of Psychiatrists, Faculty of ID, and key academics in field was undertaken. This was done to ensure that scope of research questions remains relevant to evidence-based clinical practice. This resulted in refining the research questions and by consensus, exclusion of areas such as diagnosis of ID, autism etc. The decision to exclude these areas was made as primarily, they are wider areas of enquiry and hence kept out of scope of this review. The research question hence focused on examining validated reports in few key areas, such as challenging behavior, outcomes in forensic ID, Dementia, Epilepsy, and ADHD in ID. We also attempted to examine some of the generic outcome instruments for key conditions where it was felt relevant (i.e., the tool was claimed to be validated for people with ID).
2. Search criteria: we formulated a broad-based search strategy and searched the following databases: Medline, PsycINFO, Cumulative Index to Nursing and Allied Health Literature, and Embase. We also searched for key clinical practice guidelines and textbooks relevant to ID psychiatry. The inclusion screening, long listing and data extraction was done by the first author (MK) supported by co-authors (IS, VC, JM and RS). Any disagreements were discussed as a group and reconciled with consensus.
3. Study selection: our inclusion criteria were measures which had been validated (with psychometric property data such as reliability, internal consistency, validity) and they have included ID subjects in their research reports. We focused on peer reviewed publications limited to English language only. Tools focused primarily on autism and autism spectrum disorders were specifically excluded as there is substantial literature.
4. Analysis: Following data extraction, the findings were synthesized narratively.

## Results

### Search results

**Figure 1.**
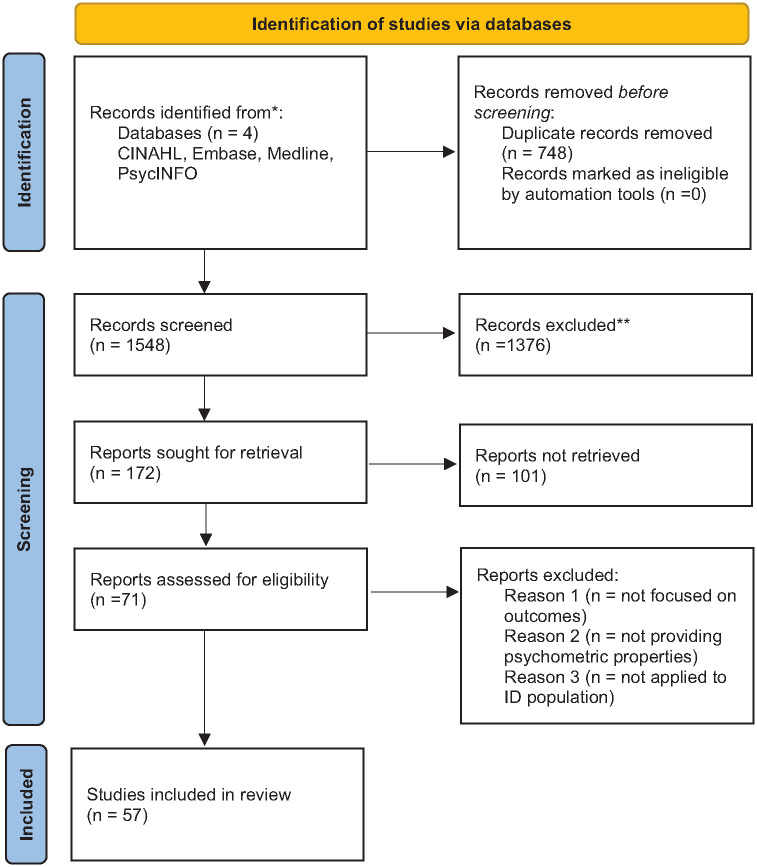
PRISMA 2020 flow diagram. *Consider, if feasible to do so, reporting the number of records identified from each database or register searched (rather than the total number across all databases/registers). **If automation tools were used, indicate how many records were excluded by a human and how many were excluded by automation tools. Source: Page MJ, et al. BMJ 2021;372:n71. doi: 10.1136/bmj.n71. This work is licensed under CC BY 4.0. To view a copy of this license, visit https://creativecommons.org/licenses/by/4.0/.

Following database searches on 7th May 2024 of Ovid-Medline, PsycINFO, Embase and CINAHL and removal of duplicates, 1,548 records were identified (Figure 1). Full texts of 57 papers were retrieved. This was aided by citation tracking and reference review from textbook and clinical guidance in relevant areas (*n* = 14). Finally, 35 papers which met eligibility criteria and included in review of which 29 were from the search and six from citation tracking/ Guidelines.

### Narrative synthesis

Several outcome measures were identified using the search strategy and these measures focused on domains of relevance to ID psychiatry. The retrieved outcome tools measured concepts such as challenging/offending behavior, and specific neurodevelopmental conditions, including ADHD, Epilepsy, alongside measures of dementia. We have arranged the results as a narrative synthesis to correspond with these concepts providing where needed a brief background to their role in people with ID.

#### Challenging behavior

“Behavior can be described as challenging when it is of such an intensity, frequency or duration as to threaten the quality of life and/or the physical safety of the individual or others and is likely to lead to responses that are restrictive, aversive or result in exclusion” (Royal College of Psychiatrists et al., 2007). Challenging behavior is a socially constructed, descriptive concept that has no diagnostic significance, and makes no inferences about the etiology of the behavior, covering a heterogeneous group of behavioral phenomena across different groups of people (The Royal College of Psychiatrists, 2013). Challenging behavior is multifactorial, but can represent a form of communication, be caused by skills deficits, be associated with psychiatric disorder or symptoms or physical illness, or develop through operant conditioning and reinforcement (Koritsas & Iacono, 2012). Due to the risks associated with challenging behavior, it is a key focus within ID psychiatry.

The British Psychological Society commissioned a study of outcome measures in ID, and through consultation they identified a list of tools, across three different challenging behavior domains: generic measures, measures of frequency and impact, and measures of quality of life (Morris et al., 2014). Using a criteria of total scores for pre- and post-interventions, the study recommended four outcome measures (Appendix 1). These were namely Health of Nations Outcome Scales- Learning Disability (HoNOS-LD), Challenging behavior interview, Behavior Problems Inventory (BPI-01) and Maslow Assessment of Needs Scales for Learning Disability (MANS-LD).

HoNOS-LD (Roy et al., 2002): is well established for use with people with ID with mental health needs, regardless of the degree of their disability. It is a clinician rated scale graded on a five-point Likert scale. The scale has been well validated and reports moderate to good interrater reliability with *k* values of (0.56–0.86) (Roy et al., 2002). It is a generic 18 item tool and does not measure individual health states. It focusses on measurement of change across domains such as behavior toward others, psychosocial problems, attention and concentration, activities of daily living, self-care etc.

Challenging Behavior Interview: (Oliver et al., 2003) is a 14-item scale aiming to assess the severity of challenging behavior in children and adults with ID, including the following domains: self-injury, verbal aggression, inappropriate vocalization, and disruption to environment. The validated tool reports excellent κ values of part 1 (0.7–0.9) and moderate for Part 2 (0.6–0.8).

Behavior Problems Inventory (BPI) (Rojahn et al., 2012): is a respondent-based behavior rating instrument for self-injurious, stereotypic, and aggressive/destructive behavior. Items are rated on a frequency and severity scale. It has good consistency and excellent inter-rater reliability (*r* = 0.76) with good test-retest reliability (*r* = 0.76). There are two versions available, (BPI-01 with 49 items, and short form with 30 items); both of which have good discriminant validity, and robust factor structures, however, they would need further adaptation. It is useful for challenging behavior at risk as an outcome for intervention studies. However, this may not be used for overall assessment of challenging behaviors, as the BPI has focused on only three domains of challenging behaviors.

Maslow Assessment of Needs Scales for Learning Disability (MANS LD): is a value driven scale quantified and adapted to the needs of ID subjects. The 19-item questionnaire utilizes a response format with a five-point scale with symbols to help the person decide on their response. Questions are focused on mapping satisfaction to well-known Maslow’s basic needs to higher need hierarchy. The MANS-LD is supplemented by an eight-item questionnaire adapted from the World Health Organization Quality of Life WHO-QOL (which is not adapted to ID specific population). The validation results show moderate to good validity and reliability data (Skirrow & Perry, 2009).

While the remit of British Psychological Society study was to measure pre and post intervention change, the remit of our scoping study is wider, so we have included other tools which meet our study criteria. Following is a narrative synthesis of other tools and outcome measures which are useful in measuring challenging behaviors in various context and are validated in ID population.

Maslow Assessment of Needs Scales (MANS) for Learning Disability, *Mini -MANS LD* (Raczka et al., 2020): reports acceptable psychometric properties, including moderate congruent validity and acceptable internal consistency (α = .74). The authors mapped the relationship between Mini-MANS LD and EQ-5D and report that it was significantly correlated with one health state. This is a significant development in quality-of-life measurement scales in people with ID and once validated further paves the way for mapping the quality-of-life data which would allow for calculations of much precise incremental cost effectiveness ratios (ICER) and hence aid in a robust decision making.

Modified Overt Aggression Scale (MOAS) (Ratey & Gutheil, 1991): has been shown to have excellent results in specific trials (κ = .65–.9). The informant rated tool considers severity of verbal aggression, aggression toward property, self-harm, physical aggression during a 1-week timeframe. However, the results are based on a small sample showing aggressive behavior, rated by two interviewers (Oliver et al., 2007). Owing to these limitations to this tool, reliability was compromised, and we have not been able to find more recent validations from this measure.

Diagnostic Assessment for Severely Handicapped (DASH II): measures mental ill health and challenging behaviors in severely intellectual disabled people. It is informant based and has 84 items rated on a three-point Likert scale. The scale shows good test-retest reliability of (0.8–0.9) but poor to moderate internal consistency (α = .53–.84). Raitasuo et al. (1999) has used DASH and the Brief Psychiatric Rating Scale (BPRS) in a Finnish study. While BPRS has been used more as a proxy measure of improvement following intervention, the study establishes the potential of BPRS to be used in people with ID. DASH is wide ranging measure and based on observation, hence it may be more appropriate for people who have communication difficulties, and hence the tool is relatively non-discriminatory. However, the study pointed that perhaps the tool in study was not effective in detecting the minimal degree of change, hence a more robust adaptation of tool is imperative.

Aberrant Behavior Checklist (ABC) (Aman et al., 1985): is one of the most widely used instruments to assess challenging behavior among children and adults with ID. This 58-item checklist has five subscales for agitation, lethargy, social withdrawal, stereotypies, hyperactivity or noncompliance and inappropriate speech. ABC has been found to have good to excellent internal consistency (Cronbach α = .8–.9) and moderate interrater reliability.

#### Outcomes in forensic ID

People with ID and forensic needs have long inpatient stays with significant health and financial implications. Outcome research has been limited to single setting service evaluations, with only a minority of multi-site outcome studies. Measuring treatment outcomes in this population is complex due to the heterogeneity of patients, and of subsequent therapies and interventions provided by the service, and there has been little agreement among services as to the domains that should be the target of outcome measurement. While studies have described the outcomes of psychological treatment programmes, such as those targeting certain index offences, few studies describe the care models followed at the service level, or the short (during admission/point of discharge), or long term (post-discharge) treatment outcomes of patients cared for within such services (Chester, 2018).

Morrissey et al (Morrissey et al., 2017) systematically examined the treatment outcome domains focused on within the empirical literature, alongside a Delphi consensus exercise investigating stakeholder views, and consultation with patients and carers. The review focused on three key outcome domains, effectiveness, patient safety and patient and carer experience. The Delphi consensus exercise conducted by the authors then provided sub-domains to each domain.

Of the 60 studies included in the review one of the most common outcomes reported domains within the literature was effectiveness. Following the consultation groups sub-domains identified were those that captured aspects of the care pathway, along with a focus on clinical symptoms, recovery and a reduction in reoffending. Related variables, such as length of stay, discharge and need for security were included, but these were not always directly correlated with clinical need. Further domains clustered around safety and the patient and carer experience were incorporated into final framework, as proxy indicators of the quality of forensic services. While the overall review provides key evidence in this area, proxy variables have dominated within the literature.

One of the other most reported outcomes is length of stay, which has limitations that it is not directly correlated with patient characteristics or their progress and can be affected by factors such as discharge placement availability. Similarly, there is wide variability in methods of reporting. Discharge placements are often used as a treatment outcome but are challenging to validate. These outcomes reflect more on health system responsiveness and impact than patient outcomes. This points to a substantial research need in this area. The review explicitly outlined that there is a gap in understanding the recovery processes at the individual level in this context and this is a relevant research need. The authors acknowledged a lack of instruments to measure outcomes in forensic ID, and a research need to develop the same. As such, there is a need to identify, develop and validate patient related outcome measures in the forensic ID population.

#### Inpatient outcome measures in ID

Another systematic review in this area (Melvin et al., 2022) has examined effectiveness, safety and experiences in inpatient settings and has reported that such services were associated with improvements in mental health for this population. The review included 16 studies which reported clinical outcomes measures: Brief Symptom Inventory, Emotional Problem Scales, Mini Psychiatric Assessment Schedules for Adults with Developmental Disabilities (mini-PAS-ADD), Health of the Nation Outcome Scale (HoNOS)-Secure, Reiss Screen for Maladaptive Behavior and Clinical Global Impressions Scale. Some of these scales are not validated in the ID population but have been used in both inpatient/forensic ID services.

Within forensic and inpatient ID settings, the two systematic reviews carried out indicated that process measures such as length of stay and select clinical outcomes were most reported. While such reviews are crucial in determining health system responsiveness and key priorities, there is a need to better understand which patient related outcome measures are more sensitive to change in ID population in both forensic and inpatient settings. There is a pressing need to map utility data so that cost effectiveness data can be gathered.

#### Dementia

Assessment of dementia can be challenging in people with ID. However, there are some scales specifically designed to diagnosing dementia.

Dementia for learning disability (DLD) scale: was developed in the 1980s in The Netherlands, and its intended use was for adults with ID. Since then, it has been used widely in Europe and in the UK, both in clinical practice as well as in research (Strydom & Hassiotis, 2003). This is completed by a family member or carer who knows the person well. It has 50 items giving two main scores, namely, cognitive scores (SCS) and social scores (SSC). While a study (Evenhuis, 1996) has reported that the DLD has a sensitivity of 100%, other studies have shown that DLD has moderate sensitivity and specificity (0.61/0.63). Despite its wide usage. DLD test–retest reliability was also moderate overall but was stronger for the cognitive subscale than the social subscale.

Rapid Assessment of Dementia in Developmental Disabilities (RADD) (Walsh et al., 2015): sensitivity to dementia in Down Syndrome for RADD exhibited high sensitivity (0.87) and specificity (0.81) in discriminating among individuals with and without dementia.

Dementia Screening Questionnaire for Individuals with Intellectual Disability (DSQIID): DSQIID is 53 item dementia screening questionnaire for people with ID. The scale reported internal consistency α = .91 inter rater consistency of 0.9 and test retest validity is 0.95. (Deb et al., 2007; O’Caoimh, 2013) The scale has a fixed cut off score which limits its applicability in people with more severe forms of ID.

Modified Cambridge Cognitive Examination with Down Syndrome (CAMCOG-DS): This modified version of the Cambridge Cognitive Scale (CAMCOG) has been adapted from Cambridge examination of health mental disorders in Elderly (CAMDEX) and has been validated in the Down Syndrome population (Hon et al., 1999). The tool reports a κ (inter rater reliability) of 0.8 for 91% of domains rated, indicating good reliability (Ball et al., 2004).

Geydes Dementia Scale for Down Syndrome (Jozsvai et al., 2018): This scale for assessing severity of dementia reports a good sensitivity and specificity of 85% and is one of the NICE recommended rating scales for assessment of dementia in Down’s syndrome.

Quality outcome measures in Dementia (QoMID): QoMID is one of few validated tools to measure quality outcomes in dementia in the ID population (Dodd et al., 2015). The tool shows robust psychometric properties. Principal component analysis (PCA) shows that QoMID tool components have good eigen values and hence factorizable. It has good face validity. The QoMID has good internal reliability (Cronbach α .84) suggesting that all domains contribute equally toward the construct of quality outcome. The authors have defined quality in a comparative manner by rating a person across domains through stages of early, mid stage and advanced dementia stages and their ease of adaptability. Assessors on QoMID rate using inclusive judgment across each domain.

#### Epilepsy

Approximately a quarter (22.2%) people with ID have epilepsy (Robertson et al., 2015). Of these, 70% are medication resistant (Doran et al., 2016). Given that epilepsy is an umbrella condition, with many causes and comorbidities, a wide range of issues need to be considered when measuring outcomes in this population (Shankar, 2023; Watkins et al., 2022) . This causes significant level of disability and premature mortality among people with ID with epilepsy (Shankar, 2023; Sun et al., 2023; Sun et al., 2022). There is a significant health related quality of life impact among people with ID (Chiang et al., 2021).

Seizures: Seizures are the hallmark symptom that defines epilepsy, and therefore the occurrence of seizures is an important outcome to measure when assessing the effectiveness of interventions. Traditionally seizure diaries have been used, commonly recording seizure types, seizure occurrence and duration, any triggers, associations with medication change or other factors such as sleep, stress and the menstrual cycle (Berg et al., 2024). In people with ID this may be captured using “easy read” diaries or be managed by a carer or family member. Data on seizures may become more automated in the future, with the increasing data on using long term ambulatory forms of EEG monitoring(Milne-Ives et al., 2023; Wang et al., 2019).

Safety issues: People with ID and epilepsy are at significant risk of harm from seizures. A good practice tool is evidence based tool is the Sudden Unexpected Death in Epilepsy (SUDEP) and Seizure Safety Checklist (Shankar et al., 2018). It allows for understanding and communication change in risk in people with ID. An evidenced patient facing digital tool EpSMon is available to support and feedback on risk change (Newman et al., 2020). Data on the consequences of seizures including seizure related injury, mortality including SUDEP, and unplanned healthcare utilization is also recommended to be collected routinely in clinical practice by the International Consortium for Health Outcome Measurement (Berg et al.) Epilepsy Outcome Set.

Drug treatment: Measuring the impact of anti-seizure medication (ASMs) is important (Doran et al., 2016; Watkins et al., 2020). There is good guidance of suitable best practice in prescribing ASMs to people with ID (Watkins et al., 2020). The use and removal of rescue medication specifically midazolam is a good indicator of improved or worse outcome (Tittensor et al., 2021).

Holistic presentation: The HONOS-LD captures seizures as a category and among other issues links it to the general presentation of the individual.

System outcomes: The purple light toolkit developed with co-production is a tool to examine service and community outcomes for people with ID and epilepsy. It is recommended by the Royal College of Psychiatrists and NHS England (“NHS-England”; Shillito et al.)

The annual LeDeR report provides insight to local mortality outcomes including epilepsy in ID. ICHOM have recommended to measure anxiety, depression, sleep and quality of life too, routinely in this population. However, there are no specific tools validated in the epilepsy population to measure these constructs, except for the ELDQOL scale.

#### Attention deficit hyperactivity disorder (ADHD)

ADHD is co-morbid in up to 20% of people with ID (Miller et al., 2020). Accurate diagnosis allows for treatment, and thus outcomes are directly linked to diagnosis. To facilitate suitable diagnosis, and by extension best outcomes, structured assessments are important.

For the general population, the Diagnostic Interview for ADHD in Adults (DIVA), a structured assessment based on DSM-5 criteria for ADHD has been developed. The DIVA has been modified to encapsulate the presentation of ADHD in people with ID (Kooij et al.). This involved providing concrete clinical examples (in child and adults) of the 18 symptom criteria. The clinical examples are common presentations described for carers and patients to see how similar their individual experience is. This includes a range of social influences such as work/school, relationships, recreational and social activities. The DIVA-ID has not been separately validated in the ID population. There is now a proposed screening tool for ADHD in people with ID (Sawhney et al., 2021). Using multiple logistic regressions, three questions were identified and proposed for screening. It needs larger-scale replication to generate generalizable results. While the DIVA is an assessment tool, from a pragmatic perspective, there is also potential to use DIVA as a proxy outcome measure.

There is significant limitation of lack of outcome measures in ADHD in ID. Conceptual domains such as response to behavioral symptoms, functional impairments (in familial, social, adaptive, emotional and occupational areas), quality of life, adaptive life skills, and executive functioning need to be evaluated comprehensively to understand the impact, this area of enquiry needs to be developed further.

#### Other key outcome measures used for mental ill health and ID

There are few validated measures, which are used across the spectrum of services and are based on clinical and diagnostic schedules. They are not specific to condition or a specific measurement domain, few of those which met criteria in our review are summarized below.

Psychiatric Assessment Schedules for Adults with Developmental Disabilities PAS-ADD & mini PAS-ADD (Moss et al., 1998): PAS-ADD is designed to be used in the community and provides cumulative scores to cluster in affective/neurotic disorder, psychotic disorders and possible organic disorder among adults with ID. The tool has a sensitivity 66% and specificity of 70%. Of the nine factors the first factor accounts for 20% of the variance and the subsequent eight factors accounted for only 4% to 8% of the variance.

Mini PAS-ADD has seven subscales for depression, anxiety, bipolar disorder, psychosis, obsessive compulsive disorder, unspecified disorders, and autism (Prosser et al., 1998). Other than the anxiety and bipolar disorder subscales, the scale shows good internal consistency and reports a good interrater reliability with κ of .74. There is report of good validity (positive predictive value owing to high specificity). An independent community sample validation reported sensitivity of 66% and specificity of 70% (Sturmey et al., 2005). Overall, both PASS-ADD and Mini-PAS ADD continue to be a reliable and valid instrument to measure psychiatric outcomes in ID.

Depression-Glasgow Depression scale for people with learning disability (Cuthill et al., 2003): This is a self-report instrument that measures depression among people with ID. It is a 20-item scale with good internal consistency (α of .9). It has good test retest reliability and interrater reliability of .98.

Glasgow Anxiety Scale for people with Intellectual Disability (GAS-ID) (Maïano et al., 2023): This is a 27-item self-rating scale to measure anxiety among people with mild ID which has reported a good test-retest reliability and good internal consistency (α of .96) (Mindham & Espie, 2003). This has been further validated in a large sample (*n* = 361) comprising of mild to moderate ID subjects and reports a robust and valid factor structure with moderate stability in anxiety cluster of symptoms over 1 year period.

Reiss Profile for Mental Retardation/ Disability (Lecavalier & Havercamp, 2004): Reiss Profile is designed to assess mental health problems in people with ID. It has 15 subscales focusing on aggressive behavior, psychosis, paranoia, depression, dependent personality disorder, avoidant disorder, and autism. The tool reports a good internal consistency (average alpha = .84), significant variability in the interrater reliability (average intraclass correlation coefficient = .52), and excellent validity (95% of the correct profiles were chosen).

Anxiety Depression and Mood Scale (ADAMS) (Esbensen et al., 2003): ADAMS is 55 item Likert scale which is completed by the informant. This purpose-built scale has been shown to have good internal consistency (α of .90 with mean subscale α of .83). ADAMS shows a good construct validity with confirmatory factor analysis showing robust factor structures.

Clinical Global impression scale (CGI) (Busner & Targum, 2007): CGI is a popular consensus scale to identify outcome measures for all three stakeholder groups (clinicians, patients, family/carers) in psychiatry. While not validated within the ID population, it is established as a baseline for overall clinical research. CGI is a brief, stand-alone assessment of the clinician’s view of the patient’s global functioning both before and after intervention. It provides an overall clinician-determined summary measure that considers all available information, including a knowledge of the patient’s history, psychosocial circumstances, symptoms, behavior, and the impact of the symptoms on the patient’s ability to function. While it effectively provides a snapshot view, and is easy to administer, it consistently lacks details.

## Discussion

The review was a scoping review with aim to identify validated measures across the ID population. The review found that there are a limited number of tools available which are validated across key areas usually relying upon clinical diagnostic schedules.

Conventionally, treatment outcomes in ID have been measured only from the clinician perspective, and this is reflected in the empirical literature. Within the ID field, there is a pressing need to develop or adapt treatment outcome measures which have excellent psychometric properties, and are tailored to the presentation of psychiatric, behavioral, physical health, and quality of life needs of this population. While clinician rated outcome ratings have predominated in ID populations and settings, their perspective on treatment outcome measures is only one part of the picture. Patient rated outcome and experience measures have been emphasized within general psychiatry yet are not routinely collected in ID settings.

Furthermore, due to the characteristics of this population and their realistic dependency needs on their family/carers through the lifetime, carer rated outcome and experience measures are another important perspective which need consideration. It outlines the need for the perspectives of a wider range of stakeholders in ID treatment outcome research.

### Challenges of developing objective outcome measurements for people with ID

There are challenges to the use of PREM/PROM within ID, which largely relate to accessibility. Many core deficits associated with ID can challenge reliable and valid self-reporting, including reading level, receptive language level, cognitive processing (recalling information, ordering information, or making comparisons), articulating a response (Chester et al., 2015). Difficulty also arises when using subjective, or abstract concepts, and negative or passive phrases (Emerson et al., 2013). In forensic ID services, which largely admit patients within the mild range of ID, average literacy attainment levels are roughly equivalent to those expected by a 6 to 7 year old (Petty et al., 2013). This is likely to reduce further still when considering patients within moderate-profound ranges of ID.

Short-term memory difficulties may prevent the person from holding questions in their memory while they decide upon an appropriate response (Mark, 2011), particularly when interpreting sentences which use complex or unusual structures. Response biases are common among people with ID; acquiescence (the tendency to say yes to questions regardless of content) and recency bias (the tendency to select the last option mentioned in multiple-choice questions, irrespective of one’s true opinion), or naysaying (saying no to every question). Some response formats are more susceptible to such biases (Mark, 2011), such as complex Likert rating scales with simpler response scales (e.g., yes, sometimes, no) being a better option. Indeed, many self-report scales are designed, or adapted specifically for people with ID, using simplified question wording and response formats, minimizing the afore described cognitive and linguistic difficulties (Emerson et al., 2013).

### Psychiatric needs of people with ID

The current policy context and direction of future of inpatient services in ID is evolving, with a growing emphasis on community treatment (The Royal College of Psychiatrists, 2013; Walton et al., 2022). However, there remains significantly higher levels of psychiatric morbidity in people with ID, which mandates that the identification and treatment of their comorbid mental health problems require specialist expertise both in generic and specialist settings (Jones et al., 2021; Walton et al., 2022). Hence, it is crucial to revisit the need for monitoring long term needs by formulating and monitoring outcome measures both for informing data driven robust approaches as well as for assisting resource allocation decisions.

### Limitations of study

Our scoping review is an attempt to map the validated outcome measures specific for ID. However, in current report in absence of clear outcome measures as defined by international consortium for health outcome measures (Berg et al.) we have interchangeably used validated measures which can be used as clinical outcome measures.

Since, this is a scoping review, our remit is very wide, and we have attempted to map the validated outcome measures or instruments which have included people with ID. However, this is limited to English language peer reviewed literature, and there may be other relevant instruments who have used more than one condition screening or dual diagnosis screen which may have been screened out of the review. Also, as scoping review entails, we have been inclusive of all study designs and measures and qualitative appraisal and risk of bias assessment has not attempted. It is recognized that 15% of people with ID have co-morbid autism. However, autism and autism spectrum disorder was excluded from the search. It was felt that this would be a topic in it’s own right where there is considerable high-quality literature already present (Brugha et al., 2015; Grzadzinski et al., 2020; Howell et al., 2021; McConachie et al., 2015; Ridout & Eldevik, 2023; Wigham & McConachie, 2014).

### Implications for research and practice

A major challenge is that in a significant proportion of people with ID, a health condition does not exist in isolation but as multiple long-term condition (Kinnear et al., 2018). Thus, while a single disease outcome tool may some relevance in treatment monitoring, a broader patient focused outcome measure would be more meaningful in capturing what matters to people with ID and their carers. At present, other than for HONOS-LD there is no such composite tool which is largely recognized, validated or used in clinical settings. This is an area which needs further research.

People with ID are at significant risk of being “left behind” in research and all attempts need to be made to be inclusive of them including in developing outcome measures (Bishop et al., 2024). Currently, nationally in the United kingdom, the NHS England has prioritized outcome measurement as policy and has a renewed focus on use of Patient Reported outcome measures (PROM) across all community mental health services (‘NCCMH’, 2023). Within the ID field, the state of outcome measurement relies mostly on process indicators at systems level, and proxy use of CROM primarily some validated measures. There is a paucity of measures which reflect the need and aspirations of ID populations. People with moderate to severe ID have been excluded from this measurement exercise, as a consequence as there are inherent limitations to rating tools which does not take alternative communication techniques. In absence of robust and representative measures designed specifically for people with ID there is a risk of replicating and adapting measures designed for general population. Participatory research should be a way forward to understand relevant outcome measurement domains (De Kuijper et al., 2023).

A challenge in clinical rated outcome measures is lack of construct validity specifically powered to detect changes across the spectrum of ID, despite sharing a common developmental platform, such as ICD-11 or DSM-V criteria. Assessment of treatment outcomes needs to move beyond the scope just symptomatic improvement, and encompass measures of adaptive functioning, quality of life, and integration/ return to functioning- latter is more relevant to ID than any other psychiatry disciplines. There is a need for development and validation of clinical outcome measures as well as developing inclusive participatory research, which addresses challenges for differently abled.

Measuring utility is essential for outcomes research because it provides a way to assess the effectiveness of healthcare interventions in terms of their impact on patients’ quality of life. Utility measures can help healthcare providers, policymakers, and researchers make informed decisions about resource allocation and treatment choices based on the outcomes that matter most to patients. The most widely accepted utility measure for allocation decision is the QALY, the data for which can be generated from EQ-5D, however, currently there are rare application of EQ5D data which is beginning to make headway (Raczka et al., 2020) have used EQ5D in part. Currently, lack of such data in ID implies that there is a limitation of using cost-impact, budget impact and cost consequence analysis and hence limiting a rational voice in resource allocation decision.

## Conclusion

This review aimed to synthesize the treatment outcome measures in field of ID. The findings highlighted a pressing need for work to adapt and validate existing measures for use within the ID population, and to design and validate measures specifically tailored to the needs of this population. The area comes with its unique sets of challenges, primarily the historical exclusion from research of those who have alternative expression or are non-verbal. There are issues with accessibility in the design of outcome measures for this population to self-report, including reading ability, comprehension, response biases, and difficulties with response scales. Furthermore, there are difficulties around validating proxy reports from family carers.

The science of outcome measurement has remained in close confines and has therefore perpetuated exclusion of research in ID outcomes (Bishop et al., 2024). The service imperative has allowed some focus on process level measures, which has helped in addressing commissioning priorities. However, in terms of its utility in value-based medicine and decision making in scientific manner, lack of robust metrices such as ICER and QALYs has remained completely elusive. There is an underserved population here who risk being left behind in the era of value-based medicine and increasing use of outcome measurement when assessing the effectiveness of healthcare interventions on individual and population levels. There are other additional constraints, the current level of evidence is mostly centred around diagnostic tools, and rarely around true outcome measurement, there is a pressing need to evolve conceptual areas which are not only truly reflective of clinical need, but also those that incorporate quality of life and adaptive functioning. In conclusion, there is urgent priority to rethink how best to design, replicate measures across ID to deliver a value-based care in an inclusive rights-based manner.

## Supplemental Material

sj-docx-1-isp-10.1177_00207640241291517 – Supplemental material for Outcome Measures in intellectual disability: A Review and narrative synthesis of validated instruments Supplemental material, sj-docx-1-isp-10.1177_00207640241291517 for Outcome Measures in intellectual disability: A Review and narrative synthesis of validated instruments by Mrityunjai Kumar, Indermeet Sawhney, Verity Chester, Regi Alexander, James Mitchell and Rohit Shankar in International Journal of Social Psychiatry

## Appendix

**Appendix 1.** Five select outcome measures considered in BAPS 2014 pilot study (28).

HONOS-LD	CBI	BPI-01	LEC	MANS-LD
*P* = .0013	*P* = .3371	*P* = .0002	*p* = .0719	*p* = .0121
